# Recurrent Metastatic Renal Cell Carcinoma Diagnosed With Endoscopic Ultrasound-Guided Fine Needle Aspiration 18 Years After Initial Surgery

**DOI:** 10.7759/cureus.32147

**Published:** 2022-12-02

**Authors:** Salman Ahmed, Manjula Garapati, Mohamad A Eloubeidi

**Affiliations:** 1 Department of Internal Medicine, Alabama College of Osteopathic Medicine, Dothan, USA; 2 Department of Pathology, Regional Medical Center, Anniston, USA; 3 Department of Gastroenterology and Advanced Endoscopy, Regional Medical Center, Anniston, USA

**Keywords:** post-nephrectomy renal cell carcinoma, endoscopy, immunohistochemistry staining, eus-fna, renal cell carcinoma (rcc)

## Abstract

Endoscopic ultrasound-guided fine needle aspiration (EUS-FNA) is an alternative approach to sample kidney lesions that is less commonly used compared to percutaneous CT or ultrasound guidance. In this case, we present a 70-year-old female who was diagnosed with metastatic renal cell carcinoma (RCC) 18 years post-nephrectomy with EUS-FNA in conjunction with immunohistochemistry. This case report supports the use of EUS-FNA in conjunction with immunohistochemistry as a robust technique that can safely and effectively diagnose recurrent renal cell carcinoma.

## Introduction

Renal cell carcinoma (RCC) is the ninth most common cancer worldwide, and it is more common among males [1]. It is the most common renal malignancy, and it accounts for 2% of all adult malignancies and 4% of new cancer cases in the United States [2]. In up to 5% of patients, local recurrence of RCC can occur within the renal fossa or at the resection margin in cases of partial nephrectomy [2]. Endoscopic ultrasound-guided fine needle aspiration (EUS-FNA) has been introduced since the early 1990s to sample mediastinal, pancreatic, and intra-abdominal lesions [3]. We recently encountered a case where metastatic renal cell carcinoma was detected within the left kidney fossa and diagnosed by endoscopic ultrasound-guided fine needle aspiration (EUS-FNA) biopsy in conjunction with immunocytochemistry. The case was diagnosed 18 years after a left nephrectomy for RCC.

## Case presentation

**Figure 1:**
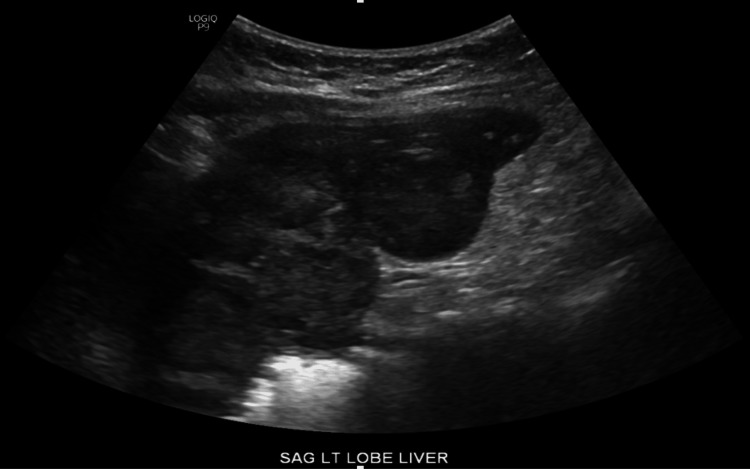
Ultrasound image of liver lesions showing hyperechoic and vascular lesions suggestive of metastatic renal cell carcinoma.

**Figure 2:**
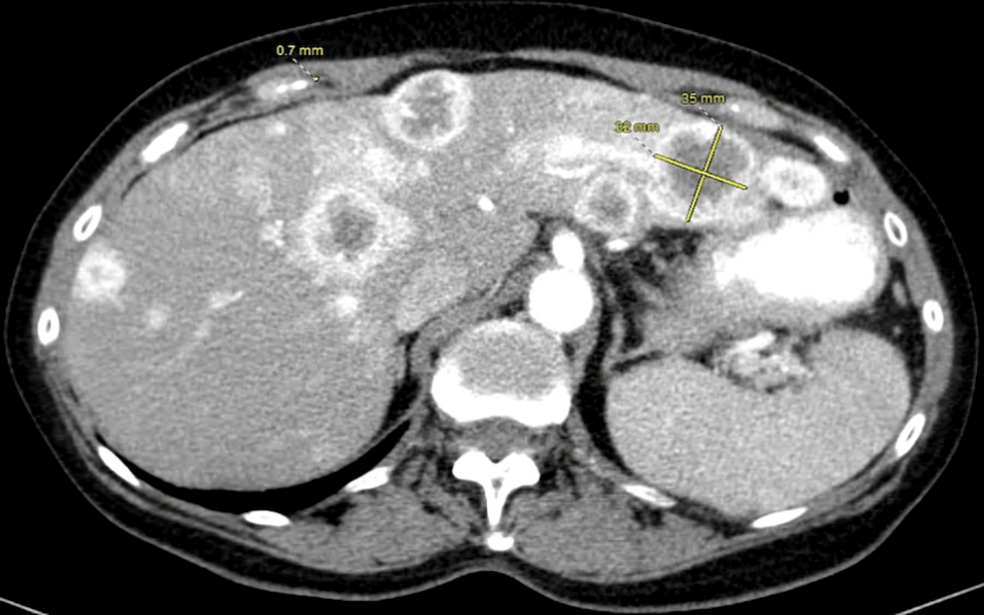
CT scan of the abdomen showing liver lesion in the lateral left hepatic lobe measuring 3.5 × 3.2 cm. CT: computed tomography

**Figure 3:**
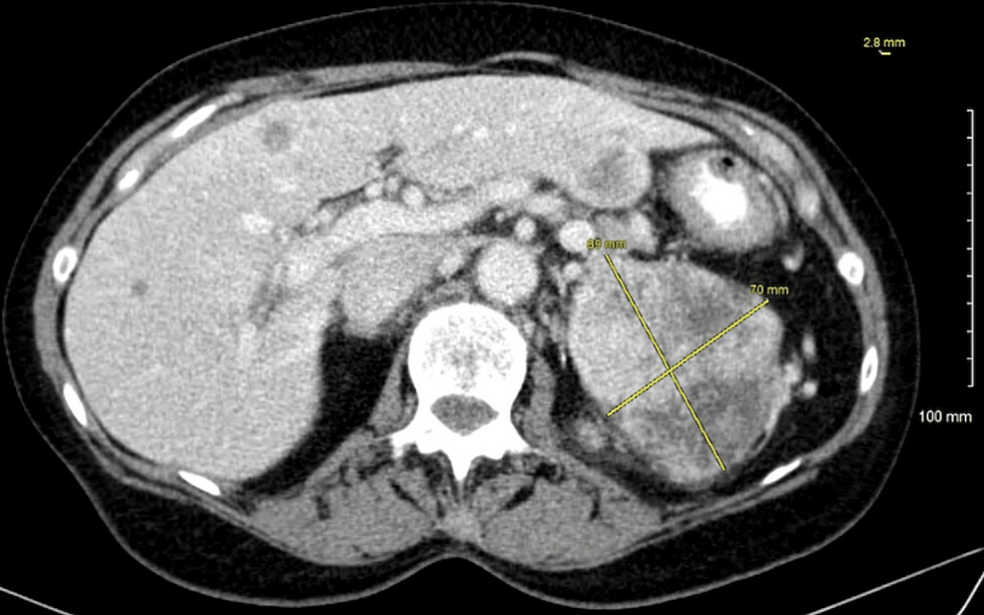
CT scan of the abdomen showing large heterogeneous soft tissue mass in the left renal bed measuring 8.9 × 7 cm. CT: computed tomography

**Figure 4:**
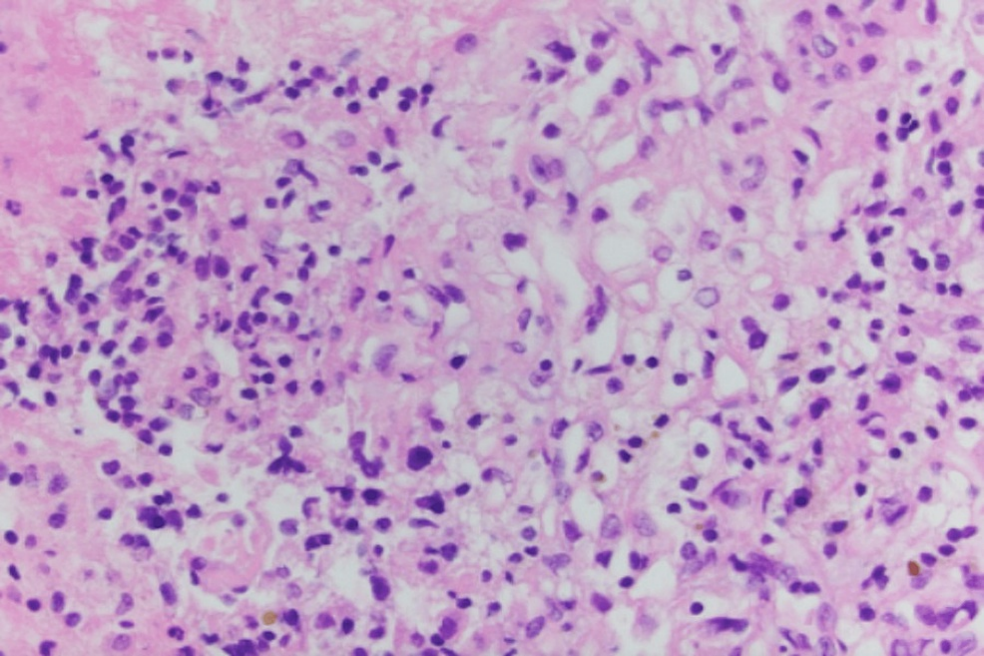
Histopathologic cell block image (H&E staining, ×40) from FNA sample of the peri-gastric mass suggestive of clear cell renal cell carcinoma. H&E: hematoxylin and eosin, FNA: fine needle aspiration

**Figure 5:**
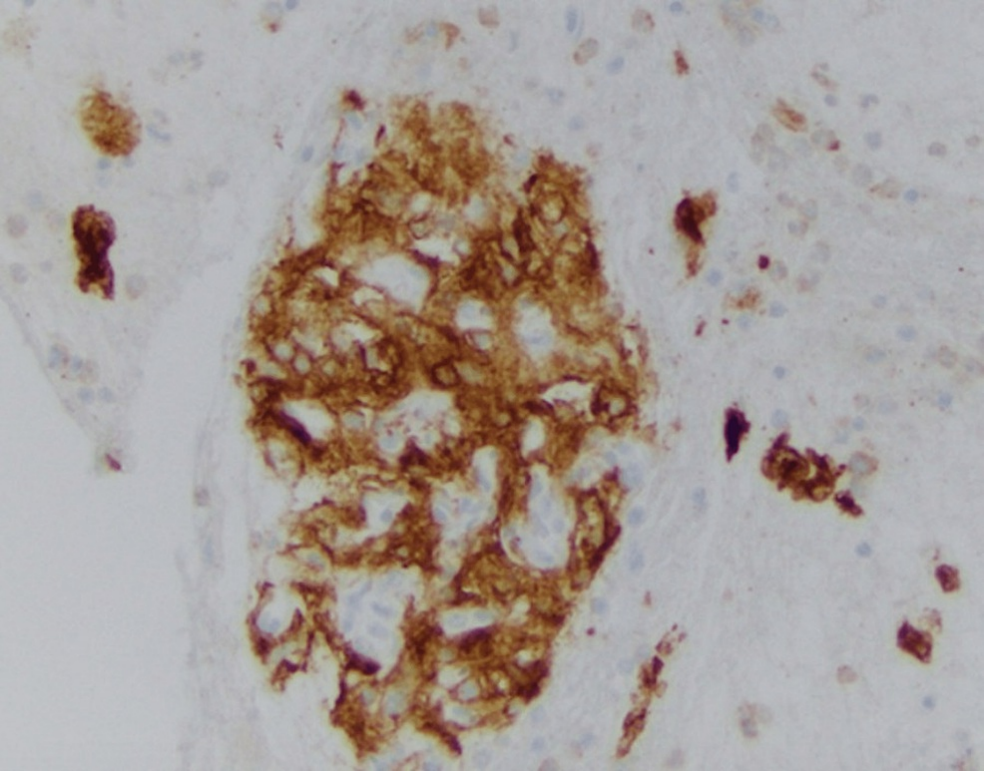
RCC-positive immunohistochemistry stain. RCC: renal cell carcinoma

**Figure 6:**
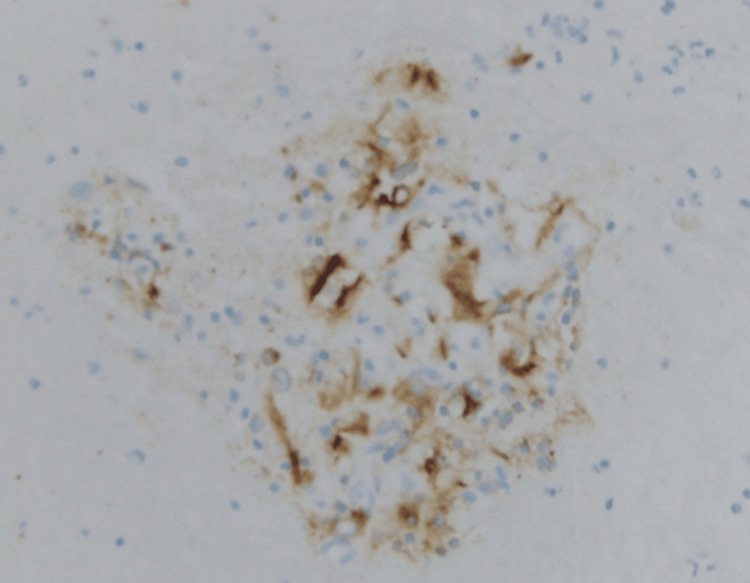
CD10-positive immunohistochemistry stain. CD10: cluster of differentiation 10

A 70-year-old white female was referred by her family to our Digestive Health practice for a complaint of 15-pound weight loss. She had undergone a left nephrectomy in 2003 for the resection of renal cell carcinoma. She had a follow-up by her surgeon for five years and was dismissed. She had a liver biopsy in 2006, which at that time showed liver fibrosis suggestive of cirrhosis, although she denied alcohol use and a family history of liver disease or gastrointestinal (GI) cancer. She denied fever, abdominal pain, and hematochezia. The patient admitted weight loss, decreased appetite, constipation, nausea, belching, and incontinence. A comprehensive physical examination was unremarkable. An ultrasound in the office revealed multiple liver lesions (Figure 1). This prompted a CT scan of the abdomen that showed numerous heterogeneously enhancing liver lesions including lesions in the lateral left hepatic lobe measuring 3.5 × 3.2 cm (Figure 2) and in the posterior right lobe measuring 2.9 × 3.7 cm. The right kidney was normal. There was a large heterogeneous soft tissue mass in the left renal bed measuring 8.9 × 7 cm (Figure 3). There were a few enlarged retroperitoneal lymph nodes. CT scan of the pelvis was unremarkable. Endoscopic ultrasound showed a 28 × 33 mm round, hyperechoic, solid mass in the left lobe of the liver. Fine needle aspiration of the mass was performed with a 22-gauge needle. There were innumerable liver lesions varying in size seen at the time with EUS consistent with metastatic disease. There was another mass in the left kidney fossa that measured 70 × 68 mm, for which fine needle aspiration was performed. Hematoxylin and eosin (H&E) stain was suggestive of clear cell renal cell carcinoma (Figure 4). Immunohistochemistry stained positive for RCC (Figure 5) and cluster of differentiation 10 (CD10) (Figure 6). The pancreas and left adrenal were normal. Upper endoscopy and colonoscopy were normal, except for mild diverticulosis in the left colon.

## Discussion

Metastatic disease develops in up to 20%-30% of patients with renal cell carcinoma following radical nephrectomy typically within six years or up to 10 years or more [2]. The most common sites of distant metastasis are the lungs, followed by the axial skeleton, lymph nodes, and liver [4]. Abdominal CT and MRI are the most common methods to stage metastatic renal cell carcinoma at primary diagnosis. Staging the tumor is important for determining the prognosis and risk of recurrence [2,4]. It is important to note that RCC surveillance guidelines are lacking with no superior strategy determined. There are, however, recognized national organizations such as the National Comprehensive Cancer Network (NCCN), the American Urological Association (AUA), and the Canadian Urological Association (CUA) that have published guidelines [5]. In up to 5% of patients, local recurrence of RCC can occur within the renal fossa or at the resection margin in cases of partial nephrectomy [2]. In addition, after a five-year disease-free period following surgery, 15% develop metastatic disease over the following decade [5]. Post-nephrectomy CT imaging is the most common imaging tool used to detect local recurrence and metastasis [2]. In some cases, tissue sampling may be required to characterize indeterminate renal lesions [6]. Traditionally, tissue sampling of renal lesions is performed by percutaneous CT or ultrasound guidance. However, endoscopic ultrasound-guided fine needle aspiration (EUS-FNA) is an alternative approach to sample kidney lesions that is less commonly used but seems to be safe and effective [6]. We previously participated in a retrospective case study of six tertiary hospitals in the United States that showed EUS-FNA is safe and feasible and has a sensitivity for malignancy of 83% for diagnosing renal cell carcinoma [6]. One benefit of EUS-FNA for renal biopsy is the ability to biopsy multiple anatomical sites during one procedure [6]. We and others have previously used EUS-FNA and immunohistochemistry for sampling various lesions in the peri-gastric organs, as well as in the mediastinum, such as the chest and abdomen [7,8]. The successful use of EUS-FNA to diagnose rare metastatic pancreatic tumors has also been shown [9]. Interestingly, the majority of metastatic pancreatic tumor types were renal cell carcinoma with metastasis up to 19 years after the primary diagnosis [9]. Furthermore, the role of endoscopic ultrasound-guided technique expands beyond the diagnosis of tissue sampling as endoscopic ultrasound-guided choledochoduodenostomy (EUS-CDS) with a fully covered self-expandable metallic stent placement in patients with distal malignant biliary obstructions has been shown to be a safe alternative technique after unsuccessful endoscopic retrograde cholangiopancreatography (ERCP) attempts [10]. In this report, we illustrate that EUS-FNA with immunohistochemistry is a robust technique that can diagnose recurrent renal cell carcinoma.

## Conclusions

We describe a patient who presented with constipation and significant weight loss. Eighteen years prior to this presentation, she underwent a left-sided nephrectomy of RCC. Endoscopic ultrasound-guided fine needle aspiration with immunochemical staining confirmed metastatic RCC. The patient was referred for appropriate therapy. This case illustrates the recurrence of RCC 18 years post-nephrectomy and that EUS FNA in conjunction with immunohistochemistry can provide tissue diagnosis.
